# Bridging the relationship between physical exercise and mental health in adolescents based on network analysis

**DOI:** 10.1002/pchj.756

**Published:** 2024-04-16

**Authors:** Mengbi Yang, Shubin Si, Kechuang Zhang, Min Xi, Weixia Zhang

**Affiliations:** ^1^ School of Mechanical Engineering Northwestern Polytechnical University Xi'an China; ^2^ Key Laboratory of Industrial Engineering and Intelligent Manufacturing, Ministry of Industry and Information Technology Xi'an, Shaanxi China; ^3^ Hospital of Northwestern Polytechnical University Northwestern Polytechnical University Xi'an China; ^4^ The Key Laboratory of Biomedical Information Engineering of Ministry of Education, Institute of Health and Rehabilitation Science, School of Life Science and Technology, Xi'an Jiaotong University, The Key Laboratory of Neuro‐informatics and Rehabilitation Engineering of Ministry of Civil Affairs Xi'an, Shaanxi China; ^5^ Department of Physical Education Northwestern Polytechnical University Xi'an China

**Keywords:** adolescents, bridge connections, mental health, network analysis, physical exercise

## Abstract

Although physical exercise has been recommended as a useful means of enhancing the mental health of adolescents, the exact mechanisms through which physical exercise plays a role are unclear. Both physical exercise and mental health are complex concepts with multiple facets, and traditional methods may constrain the manifestations of their mapping relationships. This research aimed to find the bridging connections between physical exercise and mental health. Mental health and physical exercise behaviors were assessed using the Symptom Checklist 90 (SCL‐90) and the Adolescent Physical Activity Questionnaire (PAQ‐A) in 9072 Chinese adolescents, respectively. Network analysis was utilized to construct the mental health‐physical exercise network and to analyze the relationships between individual physical exercise behaviors and mental health symptoms. Core and bridging nodes were identified based on expected influence (EI) and bridge expected influence (BEI). Gender differences were also examined. The results revealed specific and distinct pathways between physical exercise and mental health (e.g., winter sports–obsessive‐compulsive symptoms, winter sports–phobia). For both males and females, anxiety, depression, interpersonal sensitivity, ball sports, and evening activity were the most central symptoms/behaviors, reflecting their relative significance in their respective associations. The nodes with the highest BEI were obsessive‐compulsive symptoms and physical education, showing negative associations with nodes in the other community. Furthermore, in the male group, somatization and winter sports stood out as the most positive bridge nodes. Conversely, in the female group, interpersonal sensitivity and sports games were the most positive bridge nodes. These findings illuminate the pathways linking physical exercise and mental health, supporting the implementation of physical exercise in a more elaborate way.

## INTRODUCTION

Adolescence represents a key period for the development of mental disorders (Choudhury et al., 2008). Early epidemiological studies have confirmed that the incidence of mental disorders increases dramatically during this period, with negative mood changes more pronounced in girls than in boys (Costello et al., 2005; Solmi et al., 2022). There are many manifestations of psychological issues. Of them, depression and anxiety are among the leading causes of illness and disability among adolescents worldwide. Five percent of male adolescents and 6% of female adolescents aged 10–19 years experience depression (Costello et al., 2005; Jane Costello et al., 2006; Nielsen et al., 2022), whereas 5% to 10% suffer from anxiety within the same age range (Bodden et al., 2008). The prevalence rates of other psychological symptoms such as somatization, interpersonal sensitivity, phobia, and hostility in the adolescent population are also high. Unfortunately, many Chinese adolescents with psychological problems are reluctant to seek professional treatment (Shi et al., 2020). A survey among 1881 high school adolescents (aged 15–18 years) in Shantou, China found that 7.2% were seriously depressed, but that less than 5.0% sought psychological help (P. Wu et al., 2012). Moreover, it is important to note that risk‐taking behavior is a natural and necessary neurological process (Dumontheil, 2016) that is prevalent among younger individuals. While positive risk‐taking behaviors (e.g., physical exercise, making friends, etc.) are crucial for the development of physical and mental health in adolescents (Ben‐Ari, 2004), negative risk‐taking behaviors such as substance misuse and sexual behavior must be minimized to prevent negative outcomes (White et al., 2021). Some systematic reviews have concluded that physical exercise could be used as an interventional strategy to improve psychological states and reduce self‐injurious or smartphone‐addiction behaviors of students (Ali et al., 2020; Azam et al., 2020). Increasing health‐promoting behaviors related to physical exercise may be a potential strategy for avoiding the occurrence of negative risk‐taking behaviors and reducing the incidence of mental disorders (White et al., 2021).

In modern society, physical exercise is an essential component of a healthy lifestyle that individuals can undertake independently. A substantial amount of evidence suggests that regular physical exercise plays a fundamental role in maintaining healthy development in adolescents (Recchia et al., 2023; Stimpson et al., 2018; Verburgh et al., 2014). Adolescents who engage in regular physical exercise have a significantly lower risk of experiencing anxiety and depression (Pascoe et al., 2020), and certain aspects of physical exercise are negatively associated with overall symptom‐scale scores, including on the Youth Self‐Rating Insomnia Scale (YSIS), the Chinese version of the Generalized Anxiety Disorder scale (GAD‐7) and the Chinese version of the 9‐item Patient Health Questionnaire (PHQ‐9) (Chi et al., 2021; Y. S. Kim et al., 2012). In the crucial window of adolescence, active participation in physical exercise contributes to neurocognitive and psychological development and has a long‐term positive impact on future life (Fenesi et al., 2022). A prospective study showed that physical exercise is protective against mental disorders (Bell et al., 2019). Similarly, randomized controlled trials (RCTs) have also found that increasing the amount of physical exercise may reduce anxiety and depression symptom scores by using the Strengths and Difficulties Questionnaire, further supporting that physical exercise can be effective in improving mental health in adolescents (Bell et al., 2019; Larun et al., 2006). Additionally, extensive systematic reviews and meta‐analysis studies provide consistent evidence of gender differences in the positive effects of physical exercise on mental health (Do et al., 2022; Hoare et al., 2016; Rossi et al., 2021; Vaquero‐Solís et al., 2020; Wang et al., 2019). Female adolescents may reduce the time and frequency of participation in physical exercise owing to teasing or the “femininity deficit” (Halliday et al., 2019; Slater & Tiggemann, 2011; Spencer et al., 2015), suggesting the need for gender‐specific physical exercise interventions, with a particular focus on female adolescents (Do et al., 2022; Vaquero‐Solís et al., 2020).

The above studies explored the relationship between physical exercise and mental health based on the sum‐scores of psychological scales, the exact relationship between physical exercise and mental health remains unclear (Do et al., 2022; Recchia et al., 2023; Rossi et al., 2021; Vaquero‐Solís et al., 2020; Wang et al., 2019). Both physical exercise and mental health are complex concepts with multiple dimensions, and the impact of specific behaviors of physical exercise (e.g., frequency of exercise, mode of exercise, duration of exercise, etc.) on specific symptoms of adolescent mental health (e.g., anxiety, depression, somatization, etc.) is not known (Pascoe et al., 2020). Indeed, there is a lack of evidence regarding the specific interconnections between facets of physical exercise and mental health. Without this, knowledge of which symptoms of mental health are affected by physical exercise is unclear, leading to a reduced efficacy of physical exercise intervention. Only by identifying the key contact points between the specific behaviors of physical exercise and the specific symptoms of mental health can more precise and effective intervention strategies be developed. For this reason, it is important to establish a more detailed mapping of the relationship between the two concepts.

There is a clear lack of mappings between the specific behaviors of physical exercise and the specific symptoms of mental health. Network analysis offers a promising method for addressing this lack. In network analysis, items of scale are treated as nodes, and partial correlations between items are treated as edges (Fried et al., 2014, 2017). By constructing the symptom network formed by items and calculating its topographical features, network analysis is no longer limited to the overall/general correlations between physical exercise and mental health, but the associations between specific mental health symptoms and specific behaviors of physical exercise (e.g. frequency of exercise, mode of exercise, choice of time of exercise, etc.) (Bringmann & Eronen, 2018). By calculating the topographical features of symptom network, studies have revealed the core and bridging nodes of mental disorders, and both are useful in identifying targets for intervention (Cai et al., 2022; Fried & Nesse, 2014; Kaiser et al., 2021). Core nodes represent relative importance in the network structure (Borsboom & Cramer, 2013; Fried et al., 2016). Bridging nodes could establish the relationship between two domains and quantify the degree of activation/deactivation that mediates the positive/negative effects of each node on the other domain, with higher bridge values indicating a larger effect (Zainal & Newman, 2023).

In summary, the present study aims to (1) investigate the network structure linking physical exercise behaviors and mental health symptoms among adolescents, (2) compare the differences in the mental health–physical exercise network between male and female groups, and (3) identify the key connections between physical exercise and mental health. Drawing from previous studies, we hypothesize that (1) anxiety, depression, and ball sports will serve as core nodes, (2) physical education will alleviate the mental health symptom community, and (3) sports games will exhibit significant differences between male and female groups. Given the innovative nature of the analysis on the symptom–behavior network, no specific hypotheses have been formulated regarding inter‐community connections.

## METHOD

### Participants and procedure

**FIGURE 1:**
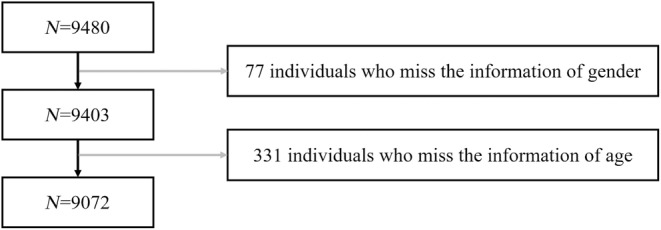
Flow chart of participant screening. Based on the original data of 9480 participants in 2020–2021, invalid data such as participants with missing information was removed, all participants included have complete data.

Our data were obtained from the National Population Health Data Center (NPHDC; https://doi.org/10.12213/11.A0031.202107.209.V1.0.) with permission. This dataset includes data on 9480 students from Shandong Province of China who were surveyed in 2020–2021 (Dong et al., 2020). After deleting invalid responses (see Figure 1 for more details), 9072 participants were analyzed (51.72% female, *M*_age_ = 16.14, *SD* = 1.24), with a response rate of 95.70%. Based on the specific geographical, demographic, and socio‐economic characteristics of 10 administrative districts in Shandong Province of China, 30 high schools and 70 middle schools were randomly chosen from each district. Three high schools and seven middle schools were then selected randomly in each district, with a minimum of 100 students per grade and at least 300 students per school. All data were collected voluntarily, anonymously and confidentially, reserved on a password‐protected website, and accessible only to direct researchers. Both parents and students provided informed consent. This study has been approved by the Ethics Committee of Shandong University (20180517) and Ethics Committee of Northwestern Polytechnical University (202302022).

## MEASUREMENT

### The Symptom Checklist 90 (SCL‐90)

The Chinese version of the SCL‐90 (Derogatis & Cleary, 1977) was used to assess the participants' mental health state. The SCL‐90 is a tool used to assess individuals’ psychological symptoms and psycho‐pathological characteristics, consisting of 90 items categorized into nine sub‐scales based on self‐reported survey questions: Somatization, Obsessive‐compulsive symptoms, Interpersonal sensitivity, Depression, Anxiety, Hostility, Phobia, Paranoia, and Psychosis. Among them, there were seven items with a focus on sleep and food that were not included in the sub‐scales and that were categorized as the sub‐scale “Sleep & eating” in this study. Each sub‐scale represents a symptom factor. Each item scored from 1 to 5 (1 = *none*, 5 = *severe*). The SCL‐90 has been widely utilized in studies measuring various mental disorders, including in medical and psychiatric inpatient and outpatient settings, clinical drug trials, and community health surveys (Maruish, 2014). The SCL‐90 has a high retest reliability (*α* = .979), as well as good discriminant and convergent validity by the Kaiser–Meyer–Olkin (KMO = .972) and Bartlett (*χ* ^*2*^ = 132256.9, *p < *.001) test (S. L. Chen & Li, 2003). Although the SCL‐90 was initially developed for usage in adults, it has also been used with adolescents. Studies have indicated that the SCL‐90 demonstrates good to excellent psychometric properties (J. Li et al., 2020; Yi et al., 2020). Nodes in the network were named from SCL1 to SCL10 according to the sub‐scales.

### The Physical Activity Questionnaire for Adolescents (PAQ‐A)

The Physical Activity Questionnaire for Adolescents (PAQ‐A) was used to evaluate adolescents' physical activity. This questionnaire is a modified version of the Physical Activity Questionnaire for Children (PAQ‐C), and its validity and reliability have been confirmed in the Chinese adolescent population (X. Li et al., 2015). Studies have shown that the PAQ‐A has good to excellent psychometric properties (Dong et al., 2020; Xu et al., 2019). The questionnaire's reliability was examined using Cronbach's alpha (*α* = .876). The 9‐item questionnaire includes 32 questions. The first item comprises 18 questions exploring the frequency of engagement in 18 different types of exercises in the preceding week. The ninth item comprises seven questions evaluating the participants' daily exercise behavior from Monday to Sunday. Additionally, items 2 to 8 include seven questions examining the intensity of exercise during various times of the day and week. The initial eight items were assessed using a 5‐point scale (1–5) (1 = never, 5 = always), where higher scores indicate higher levels of physical exercise. The last item was assessed on a 4‐point scale (1–4) (1 = none, 4 = very often) for performance on each day of the previous week (Janz et al., 2008), and the average of the total scores was used as the measure of overall weekly activity.

Previous studies have highlighted the distinct impacts of different sports categories on mental health (Asztalos et al., 2009; Chekroud et al., 2018). Specifically, while all exercise categories are linked to a reduced mental health burden compared with not exercising, popular team sports show the largest associations, followed by cycling, aerobic and gym activities (Chekroud et al., 2018). These findings indicate the necessity of further dividing sports categories for the first item in our study. Based on previous studies (Asztalos et al., 2009; Chekroud et al., 2018), we divided the 18 questions of the first item into five categories to better distinguish the effects of different physical exercise types on mental health, with questions 1, 2, 4–8, 10, and 12 as daily exercise; questions 9, 11, and 13–15 as ball sports; questions 16 and 17 as winter sports; and questions 3 and 18 as a separate category for sports games and other sports. Average values were used for each type of physical exercise. Items from 2 to 8 were named from PAQ1 to PAQ7, the measure of overall weekly activity was PAQ8, and the five categories of physical exercise were named from PAQ9 to PAQ13, accordingly. After categorizing sports, 13 items were included in the PAQ‐A scale. The results from the network analysis using merged nodes are similar to those from the analysis using unmerged nodes (as shown in the description of Figures S9 and S10 in the Supplementary Material), confirming the feasibility of our approach.

## ANALYSIS

All analyses were conducted in R version 4.3.1 (https://mirrors.ustc.edu.cn/CRAN/), including network construction, network estimation (centrality and bridging analysis), analysis of network accuracy and stability, and network comparison. First, previous research (Liu et al., 2023) utilized the graphical least absolute shrinkage and selection operator of the extended Bayesian information criterion (EBIC) in the R‐bootnet (version 1.5) package (Epskamp et al., 2018) to estimate the network and remove artificial associations. Following previous recommendations (Epskamp et al., 2018), the moderation parameter was set at 0.5 to balance sensitivity, and maximize the selection of genuine edges. SCL1 to SCL10 (the 10 symptom factors of the SCL‐90 scale) and PAQ1 to PAQ13 (the 13 items of the PAQ‐A scale) were defined as nodes. Associations of the nodes were defined as edges and were calculated by utilizing the partial correlation coefficient (Epskamp et al., 2012). By removing weakly correlated edges and retaining strongly correlated edges, a more understandable sparse network was obtained (Epskamp et al., 2012). The correlations between nodes were depicted by the color of edges (blue for positive and red for negative), with the thickness of the edges indicating the magnitude of the correlation (i.e., edge weight). Node colors represented different communities. Network visualization was facilitated through the R‐qgraph (version 1.9.2) package, which offers aesthetically pleasing layout algorithms and visualizations that enhance the comprehension of network structures and relationships (Epskamp et al., 2012).

Second, network expected influence (EI) and bridge expected influence (BEI) were calculated. Previous studies has demonstrated that EI is a more suitable index to gauge a node's significance in a mental network than other centrality indexes when negative associations exist (Bringmann et al., 2019; Epskamp et al., 2017), which determines the role that a node can play in a specific community by assessing the nature and strength of a node's cumulative influence in the network (Robinaugh et al., 2016). EI (after Z‐score normalization) was calculated by the R‐qgraph (version 1.9.2) package (Epskamp et al., 2012) and represents the sum of the weight values of all edges connected to a specific node; the higher the value of EI, the more important the node in the network. BEI is the sum of the weight values of all edges connected by a specific node to all nodes of the other community (Jones et al., 2021; Liang et al., 2022). The value of BEI was calculated based on the bridge function of the R‐networktools (version 1.5.0) package to determine the bridge connectivity between nodes. BEI is a more suitable index for identifying bridging nodes with both negative and positive connections in a network compared with other bridge centrality indexes, such as bridge strength and bridge closeness (Jones et al., 2021). Bridging nodes represent the top 20% of nodes in each community based on the absolute value of BEI. Higher values indicate a greater likelihood of activating the other community (C. Chen et al., 2022; Jones et al., 2021; Liang et al., 2022; Liu et al., 2023). Based on the above research, we considered physical exercise behavior with high negative BEI as indicating a negative association with various psychological symptoms, making it a protective factor for mental health. Conversely, physical exercise behavior with a high positive BEI may be considered a potential risk factor for mental health.

Third, in order to test whether the accuracy of the network estimation is affected by sampling variation, and whether the centrality parameters obtained from the network structure are stable and reduce the influence of individual cases, nonparametric bootstrapping (with 1000 bootstrap samples) was performed for each edge weight using the R‐bootnet (version 1.5) package (Epskamp et al., 2018). Narrower self‐bootstrap confidence intervals indicate a higher accuracy of the estimated network. Correlation stability coefficients (CS‐Cs) for EI and BEI were computed using the case‐dropping bootstrap function with 1000 bootstrap samples, considering both individual nodes and connections within the network to identify influential or key nodes. CS‐C values over 0.25 were considered acceptable, whereas CS‐C values over 0.50 were regarded as good (Epskamp et al., 2018). Based on the different sampling samples generated by the original data, bootstrapped difference tests for edge weights, nodes' EIs and BEIs were performed to examine if they significantly differed from each other (Epskamp et al., 2018).

Finally, we used the Network Comparison Test (version 2.2.1) package to assess gender differences in network edge, global strength and centrality (Van Borkulo et al., 2023). A total of 5000 permutations with Bonferroni–Holm corrections were performed to ensure the quality of the test and minimize errors from multiple comparisons (Eklund et al., 2016; Van Borkulo et al., 2023).

## RESULTS

### Network of mental health–physical exercise

**FIGURE 2:**
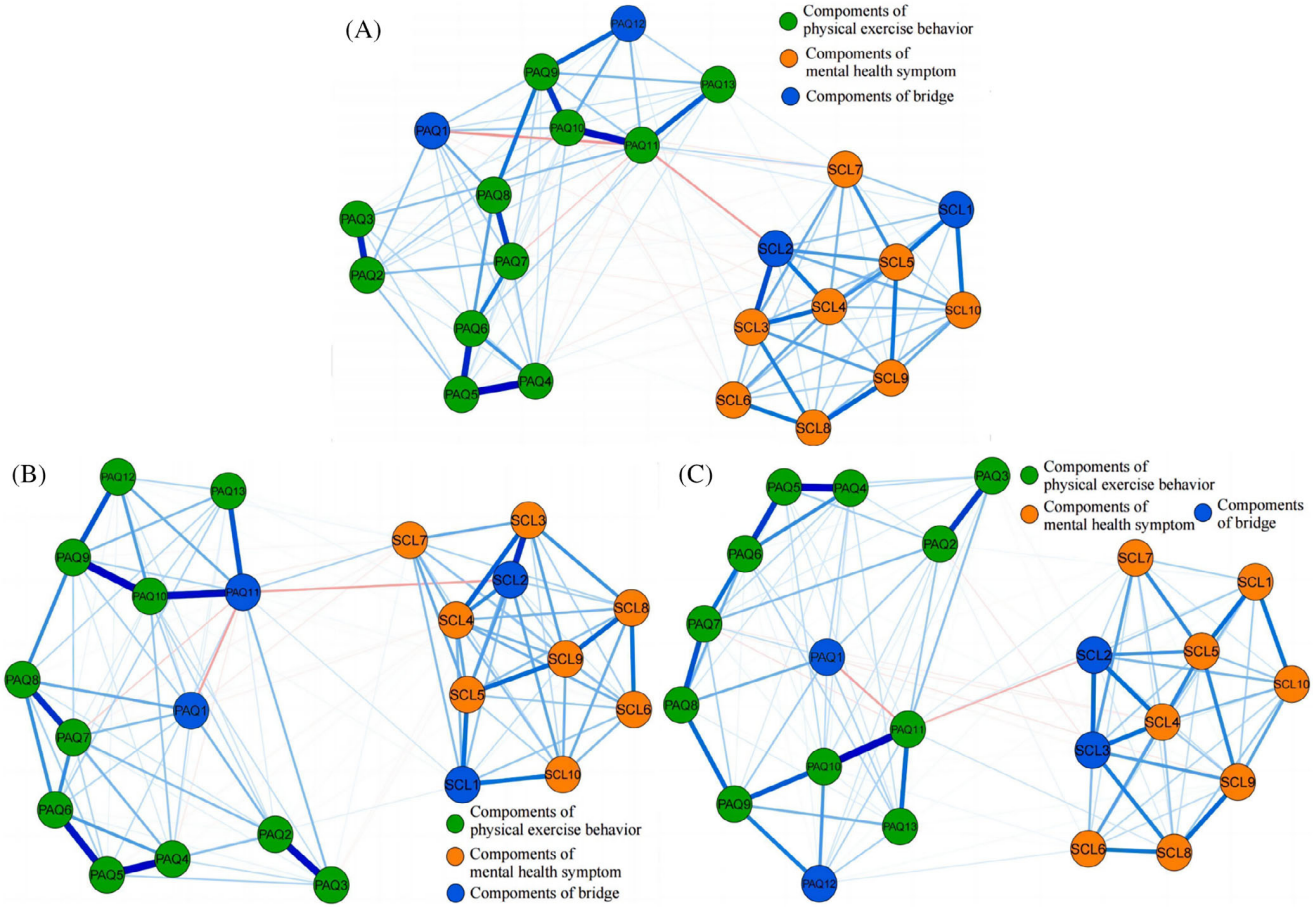
Network structures. (A) Network of all Chinese adolescents. (B) Network of male group. (C) Network of female group. Blue edges represent positive associations, and red edges indicate negative associations. Thicker edges indicate stronger correlations. Orange nodes represent the community of mental health symptoms, green nodes indicate the physical exercise community, and blue nodes represent the bridge community.

Three distinct networks related to mental health symptoms and physical exercise behaviors are illustrated in Figure 2A–C. The means, standard deviations, EI, and BEI of all nodes are shown in Table 1. The corresponding weighted adjacency matrices are presented in Tables S1–S3. Network structures without further dividing the items are also constructed (Supplementary Material Figures S9 and S10), with the main results aligning with the 13 items of PAQ‐A, further confirming the feasibility of classifying sports categories in this study.

**TABLE 1:** Descriptive statistics of nodes in the network.

Group	Node	Subscale/Item	*M*	SD	EI^a^	BEI^b^
All, *n* = 9072	SCL1	Somatization	1.430	0.574	0.872	0.074
	SCL2	Obsessive‐compulsive symptoms	1.747	0.692	0.839	−0.127
	SCL3	Interpersonal sensitivity	1.613	0.689	1.058	−0.058
	SCL4	Depression	1.556	0.683	1.085	−0.071
	SCL5	Anxiety	1.524	0.657	1.176	0.000
	SCL6	Hostility	1.466	0.641	0.844	0.042
	SCL7	Phobia	1.441	0.627	0.703	0.033
	SCL8	Paranoia	1.481	0.627	0.977	0.038
	SCL9	Psychosis	1.457	0.591	1.015	0.023
	SCL10	Sleep & eating	1.506	0.622	0.874	0.002
	PAQ1	Physical education	2.747	0.996	0.465	−0.056
	PAQ2	Morning activity	1.897	1.042	0.802	−0.004
	PAQ3	Physical exercise conducted during the midday period	1.748	0.952	0.623	−0.015
	PAQ4	Afternoon activity	1.915	1.177	0.908	−0.013
	PAQ5	Evening activity	1.866	1.138	1.051	0.010
	PAQ6	Weekend activity	2.058	1.165	0.999	0.015
	PAQ7	Free‐time weekly activity	2.234	1.047	0.797	−0.038
	PAQ8	Overall weekly activity	2.126	0.714	0.851	−0.002
	PAQ9	Fitness routine	2.142	0.835	1.024	−0.031
	PAQ10	Ball sports	1.679	0.906	1.137	−0.017
	PAQ11	Winter sports	1.350	0.860	0.793	0.031
	PAQ12	Sports games	1.910	1.184	0.697	0.052
	PAQ13	Other sports	1.808	1.202	0.697	0.024
Male, *n* = 4380	SCL1	Somatization	1.407	0.581	0.878	0.068
	SCL2	Obsessive‐compulsive symptoms	1.699	0.699	0.842	−0.119
	SCL3	Interpersonal sensitivity	1.587	0.694	1.076	−0.041
	SCL4	Depression	1.501	0.670	1.072	−0.033
	SCL5	Anxiety	1.480	0.652	1.111	0.003
	SCL6	Hostility	1.447	0.645	0.816	0.010
	SCL7	Phobia	1.378	0.611	0.757	0.054
	SCL8	Paranoia	1.480	0.642	0.984	−0.007
	SCL9	Psychosis	1.445	0.604	1.030	0.006
	SCL10	Sleep & eating	1.493	0.642	0.887	0.000
	PAQ1	Physical education	2.877	1.047	0.480	−0.037
	PAQ2	Morning activity	2.067	1.109	0.802	−0.022
	PAQ3	Physical exercise conducted during the midday period	1.868	1.023	0.628	−0.009
	PAQ4	Afternoon activity	2.062	1.255	0.857	−0.010
	PAQ5	Evening activity	2.016	1.214	1.055	0.010
	PAQ6	Weekend activity	2.204	1.220	0.972	0.000
	PAQ7	Free‐time weekly activity	2.374	1.103	0.827	−0.030
	PAQ8	Overall weekly activity	2.190	0.742	0.895	−0.003
	PAQ9	Fitness routine	2.235	0.918	1.039	−0.030
	PAQ10	Ball sports	1.901	1.005	1.184	−0.015
	PAQ11	Winter sports	1.438	0.987	0.774	0.034
	PAQ12	Sports games	1.983	1.275	0.679	0.025
	PAQ13	Other sports	1.950	1.338	0.663	0.027
Female, *n* = 4692	SCL1	Somatization	1.452	0.566	0.848	0.066
	SCL2	Obsessive‐compulsive symptoms	1.791	0.681	0.848	−0.106
	SCL3	Interpersonal sensitivity	1.638	0.684	1.039	−0.078
	SCL4	Depression	1.608	0.692	1.123	−0.065
	SCL5	Anxiety	1.566	0.658	1.218	0.000
	SCL6	Hostility	1.483	0.636	0.855	0.056
	SCL7	Phobia	1.500	0.636	0.703	0.045
	SCL8	Paranoia	1.483	0.612	0.960	0.044
	SCL9	Psychosis	1.468	0.579	0.966	0.008
	SCL10	Sleep & eating	1.518	0.602	0.857	0.000
	PAQ1	Physical education	2.626	0.929	0.434	−0.061
	PAQ2	Morning activity	1.739	0.948	0.771	0.008
	PAQ3	Physical exercise conducted during the midday period	1.636	0.866	0.588	−0.023
	PAQ4	Afternoon activity	1.777	1.081	0.957	−0.016
	PAQ5	Evening activity	1.727	1.043	1.031	0.003
	PAQ6	Weekend activity	1.921	1.094	1.003	0.025
	PAQ7	Free‐time weekly activity	2.103	0.974	0.767	−0.038
	PAQ8	Overall weekly activity	2.065	0.681	0.809	−0.004
	PAQ9	Fitness routine	2.056	0.740	1.018	−0.025
	PAQ10	Ball sports	1.473	0.745	1.052	0.002
	PAQ11	Winter sports	1.268	0.712	0.831	0.019
	PAQ12	Sports games	1.841	1.089	0.711	0.066
	PAQ13	Other sports	1.675	1.042	0.707	0.016

^a^ The expected influence (EI) value of the node index from the network, which was used in each group.

^b^ The bridge expected influence (BEI) value of the node index from the network, which was used in each group.

Figure 2A displays the network structure for Chinese adolescents. In this network, a total of 43 edges (33.08%) had a nonzero value out of a possible 130 edges. The strongest positive edge was between winter sports and phobia (PAQ11–SCL7, weight = 0.058). Conversely, the strongest negative relationship was between winter sports and obsessive‐compulsive symptoms (PAQ11–SCL2, weight = −0.095), indicating that Winter sports had the strongest positive correlation with phobia and the strongest negative correlation with obsessive‐compulsive symptoms in the context of physical exercise and mental health.

In the network for the male group (Figure 2B), 36 out of the 130 possible edges (27.69%) were observed. Winter sports–phobia (PAQ11–SCL7, weight = 0.063) and winter sports–obsessive‐compulsive symptoms (PAQ11‐SCL2, weight = −0.095) were identified as the strongest positive and negative edges, respectively. For the group of female Chinese adolescents (Figure 2C), 41 significant edges were identified (31.54%) out of the 130 possible edges. The strongest positive association was observed between Winter sports and Phobia (PAQ11–SCL7, weight = 0.039), while the strongest negative association was observed between winter sports and obsessive‐compulsive symptoms (PAQ11–SCL2, weight = −0.074).

**FIGURE 3:**
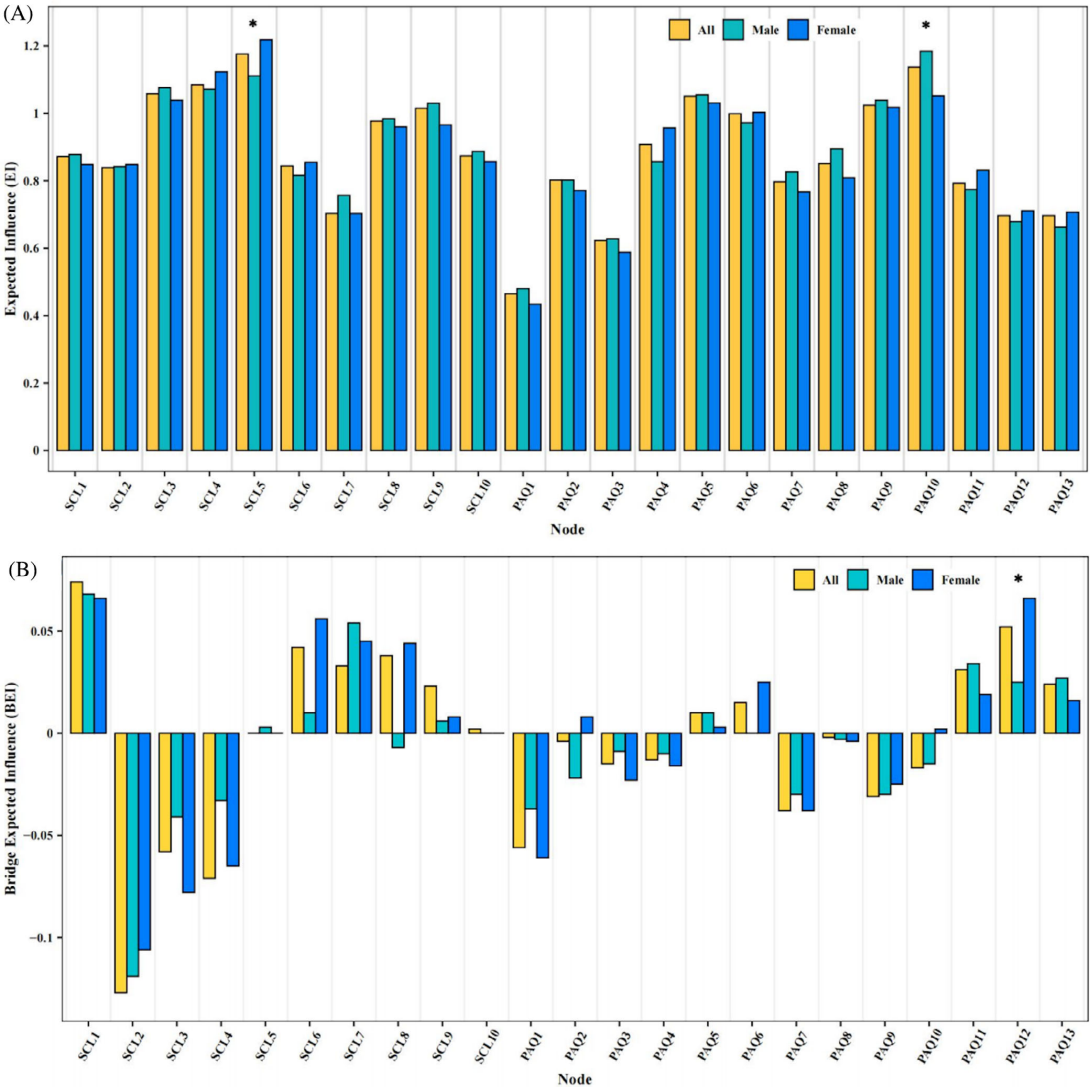
Centrality index. (A) The expected influence (EI) value of each node among all Chinese adolescents, the male group, and the female group. (B) The bridge expected influence (BEI) value of each node among all Chinese adolescents, the male group, and the female group. Bar charts for EI and BEI use yellow for all Chinese adolescents, green for the male group, and blue for the female group. An asterisk “*” at the top indicates a gender difference in the EI or BEI attribute values in the Network Comparison Test (NCT).

In Figure 3A and Table 1, anxiety (SCL5), ball sports (PAQ10), depression (SCL4), interpersonal sensitivity (SCL3), and evening activity (PAQ5) were the core nodes with the highest EI among three networks. Figure 3B and Table 1 show the nodes BEI in the mental health‐physical exercise network. The nodes with the top 20% of absolute BEI values for both the mental health community and the physical exercise community were seen as bridging nodes in the network. The bridging nodes of the three networks exhibit both similarities and differences. Notably, obsessive‐compulsive symptoms (SCL2) and physical education (PAQ1) were identified as bridging nodes in their respective communities and both had negative BEI values. In the two networks constructed from all Chinese adolescents and male adolescents, the other bridging node in the mental health community was somatization (SCL1), which had a positive BEI value, whereas interpersonal sensitivity (SCL3) had a negative BEI in the female group. In contrast, the bridging node of the network constructed from female adolescents in the physical exercise community was consistent with that of the network constructed from all Chinese adolescents. Another node with a positive BEI was sports games (PAQ12), while in the male group winter sports had a positive BEI (PAQ11).

## NETWORK ACCURACY AND STABILITY

In Figure S1, the relatively narrow bootstrapped 95% confidence intervals indicate that the accuracies of the three distinct networks are robust and can be trusted. Among the three networks, the CS‐Cs for EI and BEI were calculated to be 0.75, which means that the EI and BEI are still correlated with the original data after discarding 75% of the data (Figure S2). According to previous studies, when EI and BEI surpass the recommended threshold, the centrality indices are more reliable in the network structure. Furthermore, the nonparametric bootstrap analysis revealed that the majority of the comparisons among edge weights, EI, or BEI were statistically significant, as demonstrated in Figures S3–S6.

## NETWORK COMPARISON

When comparing the networks of adolescents of different genders, there were noticeable deviations in global strength (male participants had a network strength of 10.15, and female participants had a network strength of 10.05, *p* = .036) and network structure invariance (*M* = 0.112, *p < *.001), see Supplementary Material Figures S7 and S8. To interpret these outcomes, we computed the statistical distinctions in edge level between male and female participants by utilizing the Bonferroni–Holm correction. Statistical differences in EI and BEI values between the two groups were also analyzed. There was one edge between the two communities that was statistically significant: Psychosis and physical education (SCL9–PAQ1) (male: 0.00; female: −0.029; *p = *.025). Significant gender‐based differences in EI values were discovered for two specific nodes: anxiety (SCL5) (male: 1.111; female: 1.218; *p = *.009) and ball sports (PAQ10) (male: 1.184; female: 1.052; *p = *.014). Additionally, there was a significant difference between males and females in BEI for sports games (PAQ12) (male: 0.025; female: 0.066; *p* = .028).

## DISCUSSION

To the best of our knowledge, this study represents the first attempt to investigate the interactions between facets of physical exercise and mental health among Chinese adolescents from a network perspective. Our exploration of behaviors‐to‐symptoms enhanced the existing literature by identifying their specific associations, such as the link between winter sports and phobia, as well as that between winter sports and obsessive‐compulsive symptoms. Five core nodes were identified:anxiety, depression, interpersonal sensitivity, ball sports and evening activity. Notably, physical education surfaced as the behavior with the most significant negative impact on mental health symptoms. Gender differences were evident in the network, with winter sports affecting the mental health symptom community for male adolescents, while sports games had a similar impact on female adolescents. These findings contribute to our understanding of the link between physical exercise and mental health, shedding light on potential preventive and interventive strategies.

## RELATIONSHIPS BETWEEN PHYSICAL EXERCISE BEHAVIORS AND MENTAL HEALTH SYMPTOMS

Our analyses uncovered specific associations between physical exercise behaviors and symptoms of mental health, potentially reflecting the impact of China's National Fitness Program on the well‐being of Chinese adolescents. The strongest positive and negative connections were consistent across all three network structures, with a positive connection between winter sports and phobias, and a negative connection between winter sports and obsessive‐compulsive symptoms. Corroborating past studies on the benefits of winter sports (P. Wu et al., 2023; X. Wu et al., 2021), this study examines the impact of China's efforts to promote national participation in winter sports on the mental health of adolescents in the context of the Beijing Winter Olympics. Winter sports carry a higher risk of injury compared with most forms of exercise (Yang et al., 2021), which may make adolescents feel fearful before attempting them (Ling et al., 2022). However, this behavior can also stimulate excitement and challenge. This response is not necessarily indicative of pathological fear or anxiety (Håkansson et al., 2022). It may promote the release of endorphins, improve mood, and reduce stress. This diversion of attention from negative symptoms, such as phobia, anxiety, and obsessive‐compulsive symptoms, can have positive effects on mental health (Ainsworth & Sallis, 2022; White et al., 2021).

## EXPECTED INFLUENCE

The core nodes remained unchanged regardless of whether all Chinese adolescents or specific genders were analyzed, supporting our first hypothesis. Results on EI value indicated that Anxiety (SCL5), Depression (SCL4) and Interpersonal sensitivity (SCL3) had higher centrality values than other nodes, suggesting that these nodes play the most important role in activating and maintaining the psychopathology network of mental health. This finding aligns with prior studies on a cohort of American children and adolescents, which observed a significant association between symptoms of depression and anxiety, with these symptoms being the most prevalent indicators of development of mental health problems (McElroy et al., 2018). Additionally, other research has reported that adolescents have a greater incidence of interpersonal sensitivity, depression, anxiety, and hostility, compared with other indications of mental health symptoms (Y. Kim, 2003). A possible explanation for the core role of these three symptoms might be that interpersonal sensitivity is one mechanism through which anxiety disorders promote later depression, contributing to high comorbidity rates (Starr et al., 2014).

Regarding facets of physical exercise, ball sports and evening activity were the central nodes within the network. This finding contributes to the comprehension of mental health among adolescents, particularly in light of the uncertain mechanisms of the benefits of physical exercise to date. The prominence of ball sports and evening activities may be attributed to the heavy academic pressure faced by Chinese high school students. Prolonged sedentary time in the classroom can negatively affect their physical and mental health (Rodriguez‐Ayllon et al., 2019). Many students choose to exercise after school or at night (Saidi et al., 2020). This not only helps them unwind and provides enjoyment but also enhances the quality of their sleep and life, improving mental health (Rosa et al., 2021). Ball games are popular among youths because they foster social integration (Coalter, 2007; McAuley et al., 2000) and emotional development (Rosa et al., 2021). Team sports have been found to be more effective than individual sports in improving socialization and mental health (Andersen et al., 2019). The significance of nodes underscores the need to examine different sports categories (Chekroud et al., 2018).

## BRIDGE EXPECTED INFLUENCE

The BEI measures the interconnection between communities, with higher values indicating a greater likelihood of activating the other community (C. Chen et al., 2022; Jones et al., 2021; Liang et al., 2022; Liu et al., 2023). Breaking a bridging node could deactivate the spread of influence and decrease co‐occurrence (Jones et al., 2021). Within the physical exercise community, physical education had the lowest value of BEI, indicating that the proper intensity of physical exercise in physical education classes is protective for the healthy development of adolescents, as previous studies have shown that nodes with a higher negative BEI can be considered protective factors for mental health (Liu et al., 2023). This finding is consistent with previous cross‐sectional and prospective studies (Bell et al., 2019; Fenesi et al., 2022) suggesting that physical exercise is beneficial for mental health. By identifying the BEI, our study further suggests that targeting physical education may be more effective than other behaviors of physical exercise in reducing mental problems, which has clinical implications. For schools and teachers, prioritizing the daily physical education curriculum and ensuring a proper intensity of physical exercise during classes is vital (Valois et al., 2004). Meanwhile, students are encouraged to use the opportunity of the physical education curriculum to alleviate mental disorders (Burchartz et al., 2022; Sanders et al., 2019).

In the network of all Chinese adolescents and the female group, sports games (i.e. chasing and playing during class time) was found to have a more significant impact than other physical exercise behaviors in disrupting connections between networks based on BEI values. Three possible explanations are suggested. First, classrooms and corridors are not spacious, making accidents and spatial disputes more likely. Second, not all adolescents enjoy chasing and playing, which may result in feelings of exclusion or discomfort and trigger conflict. Third, because adolescents are less adept at managing their emotions, chasing and jostling may worsen their emotions. Reducing the frequency of conflicts among adolescents during school hours may contribute to maintaining their mental health (Slater & Tiggemann, 2011). For males, the physical exercise community in the network exhibited the highest positive values of BEI for winter sports. Winter sports are exciting and risky, with males being more inclined than females to participate. Although winter sports carry a higher risk of injury compared with most forms of exercise (Yang et al., 2021), this behavior can stimulate excitement and challenge. Proper participation in winter sports is warranted in male adolescents.

Within the community of mental health, obsessive‐compulsive symptoms and interpersonal sensitivity had the highest negative values of BEI. This indicates that these two components of mental health symptoms have stronger negative connections with physical exercise behaviors. They might be more susceptible to the physical exercise community. Somatization had the highest positive BEI, which was identified for the first time in this study according to the current mental health–physical exercise network. Possible explanations for this phenomenon include the fact that somatization is a psychological symptom strongly influenced by other psychological factors (Bekhuis et al., 2015; Busch, 2014). Physical exercise may indirectly reduce the symptoms associated with somatization by impacting other psychological symptoms, making it less susceptible to the effects of physical exercise. In contrast to previous studies, which examined the overall relationship between physical exercise and mental health (Bell et al., 2019; Do et al., 2022), our results further explore their interconnections on a more granular level. Targeting interventions addressing these connecting symptoms may reduce the risk of worsening mental health levels in adolescents.

## GENDER COMPARISONS IN THE MENTAL HEALTH–PHYSICAL EXERCISE NETWORK

The analysis revealed some significant gender differences in the overall network structure, global strength, cross‐community edges, and centrality of EI and BEI. These findings are consistent with numerous studies that suggest that gender differences should be considered in clinical interventions for adolescent mental health (Do et al., 2022; Halliday et al., 2019; Rossi et al., 2021; Vaquero‐Solís et al., 2020). Our study found that the connection between psychosis and physical education was significantly weaker in female Chinese adolescents than in males. Female adolescents may be more susceptible to stereotypes and other factors that make them less physically active on a daily basis than boys (Halliday et al., 2019; Slater & Tiggemann, 2011; Spencer et al., 2015). As a result, physical exercise may not be a priority for their emotional regulation.

Despite both male and female networks revolving around anxiety and ball sports, the EI of the anxiety node was significantly lower in the male network than in the female network, while the EI of ball sports was considerably higher in the male network than in the female network. Research has shown that male adolescents engage in physical exercise more frequently than female adolescents, particularly regarding vigorous sports such as ball sports (Hoare et al., 2016; Vaquero‐Solís et al., 2020). Moreover, girls scored lower and higher than boys on resilience and self‐reflection, respectively (Skrzypiec et al., 2014), suggesting that girls are more likely to experience a self‐imposed dilemma that leads to anxiety, which in turn hinders their engagement in physical exercise (Skrzypiec et al., 2014).

Sports games showed a significant gender difference in BEI, with females having a higher value than males, suggesting that chasing and playing during recess has a greater negative impact on the psychological state of female students. This could be attributed to the perception that female students are typically expected to engage in more feminine or less physically demanding sports compared with male students, such as dancing (Halliday et al., 2019), and may not be able to accept the frequent running and playfulness during breaks. Additionally, adolescent boys may tease girls during play, further exacerbating girls' aversion to such behavior (Best & Ban, 2021; Halliday et al., 2019). Gender‐specific physical activity interventions are needed, with a particular focus on stereotypical beliefs.

## LIMITATIONS AND FUTURE DIRECTIONS

It is important to acknowledge some limitations in the study. First, the cross‐sectional design does not allow for an examination of causal relationships among different symptoms or behaviors. Future research is needed to determine whether physical exercise behaviors influence or are influenced by symptoms of mental health, or if external situations can elicit both symptoms and exercise behaviors. Second, self‐report approaches for evaluating mental health and physical exercise behavior in adolescents may produce biased results and misinterpretations, potentially compromising the accuracy of the analysis. Further research is necessary to integrate self‐reported data with objective measures (such as biometric data) or third‐party reports (such as those from family members) to use mixed‐method approaches that complement quantitative data with qualitative insights. Third, the networks linking mental health and physical exercise reflect between‐subject effects on a group level, which may not capture within‐person processes. To enhance understanding of mental health–physical exercise relationships within an individual, idiographic network analysis using intensive longitudinal data may be essential.

Despite the limitations above, the present study is the first network analysis of physical exercise and mental health in adolescents. Identifying central nodes and bridging nodes within the network model has possible clinical significance, as targeting these nodes may contribute to protection of mental health and improve the efficacy of physical exercise intervention (Kaiser et al., 2021). The PAQ‐A covers a broad range of measures, including various categories and frequency of exercise over different time periods. Based on the findings in this paper, further research should be conducted to refine the relevant measurement instruments for potential key contact points, such as physical education, ball sports, winter sports and sports games. This can be achieved by further refining the segmentation of sports types, sports frequencies, sports intensities, etc., to identify more detailed and precise influences and key contact points.

## CONCLUSION

The complex interconnections between physical exercise and mental health are highlighted in our study. By identifying core and bridging nodes, the study offers practical implications for addressing adolescent mental health through physical exercise. These findings can benefit families, schools, and governments in the development of relevant initiatives, especially regarding improving the frequency and intensity of physical exercise. Furthermore, results on cross‐community edges revealed that China's robust promotion of winter sports enables adolescents to break away from the classroom and improve their physical and mental health. Drawing from the insights gained from network analysis on gender differences, we recommend implementing gender‐specific physical activity programs and strategies for Chinese adolescents as a preventive measure to safeguard mental health.

## FUNDING INFORMATION

This work was supported by the National Natural Science Foundation of China (72171193); the research project of China Youth & Children Research Association (2024B43); the Special funds for the Construction of World‐Class Universities (disciplines) (23GH030635); the Education Reform research of Northwestern Polytechnical University (23GZ13163); and Science and Technology Innovation Team of Shaanxi Provincial (2024RS‐CXTD‐28).

## CONFLICT OF INTEREST STATEMENT

The authors declare no conflicts of interest.

## Supporting information


**Data S1.** Supplementary Information.

## Data Availability

The data that support the findings of this study can be found online at https://doi.org/10.12213/11.A0031.202107.209.V1.0 (Shandong University. Adolescent Health Theme Database. National Population Health Data Center (NPHDC)).
