# Supplementary material for: Bridging the relationship between physical exercise and mental health in adolescents based on network analysis
Source: Psych J. 2024 Apr 16;13(5):835–48. doi: 10.1002/pchj.756 (PMC11444729; doi:10.1002/pchj.756)
Supplement: Supplementary file 1 — Data S1. Supplementary Information. [file PCHJ-13-835-s001.docx]

**Supplementary material**


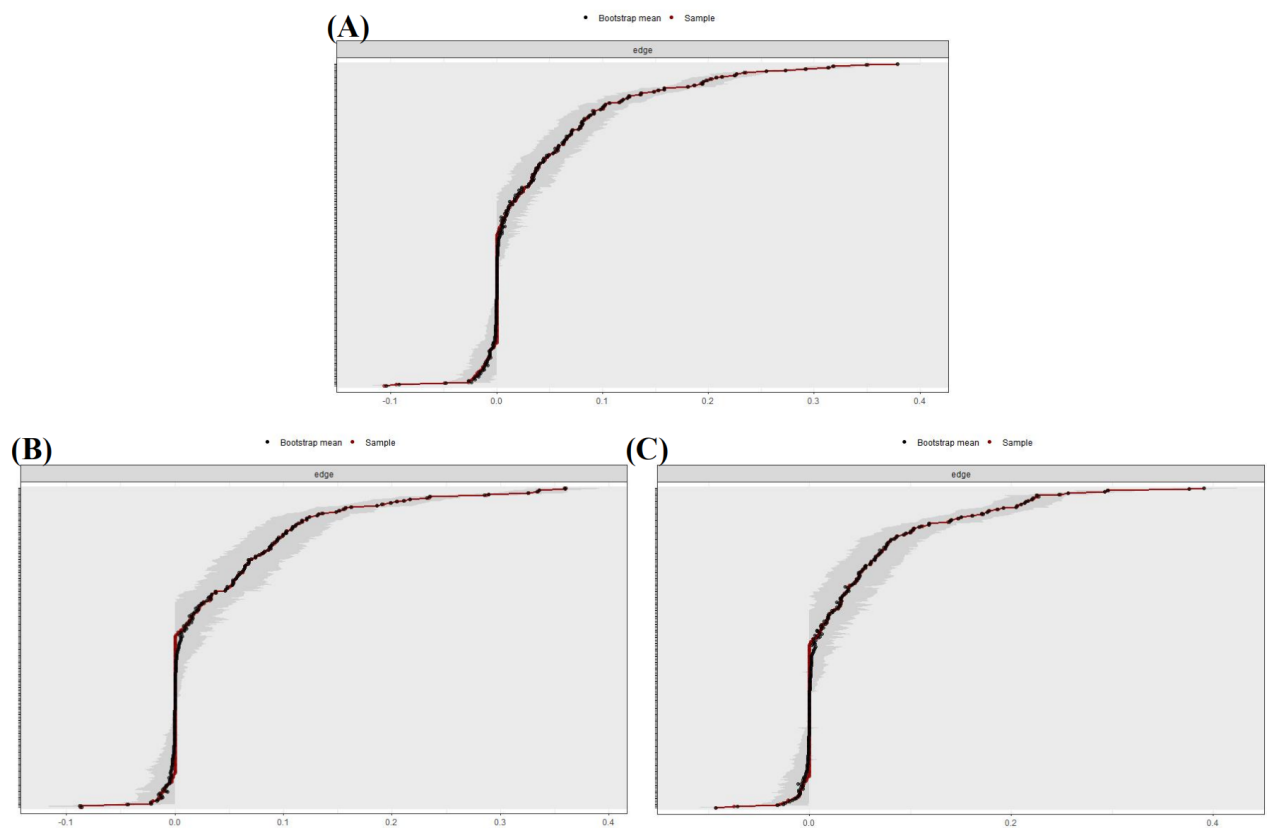


Figure S1. Accuracy estimations of all edge weights using the non-parametric bootstrapping method. (A) indicates all Chinese adolescents. (B) indicates male group. (C) indicate female group.

*Note:* Narrower 95% confidence intervals indicate reliable accuracy. The red line represents the edge, as estimated in the sample. The grey indicates 95% bootstrapped confidence interval. The x-axis represents the edges, while specific edges are denoted along the y-axis by the grey lines.


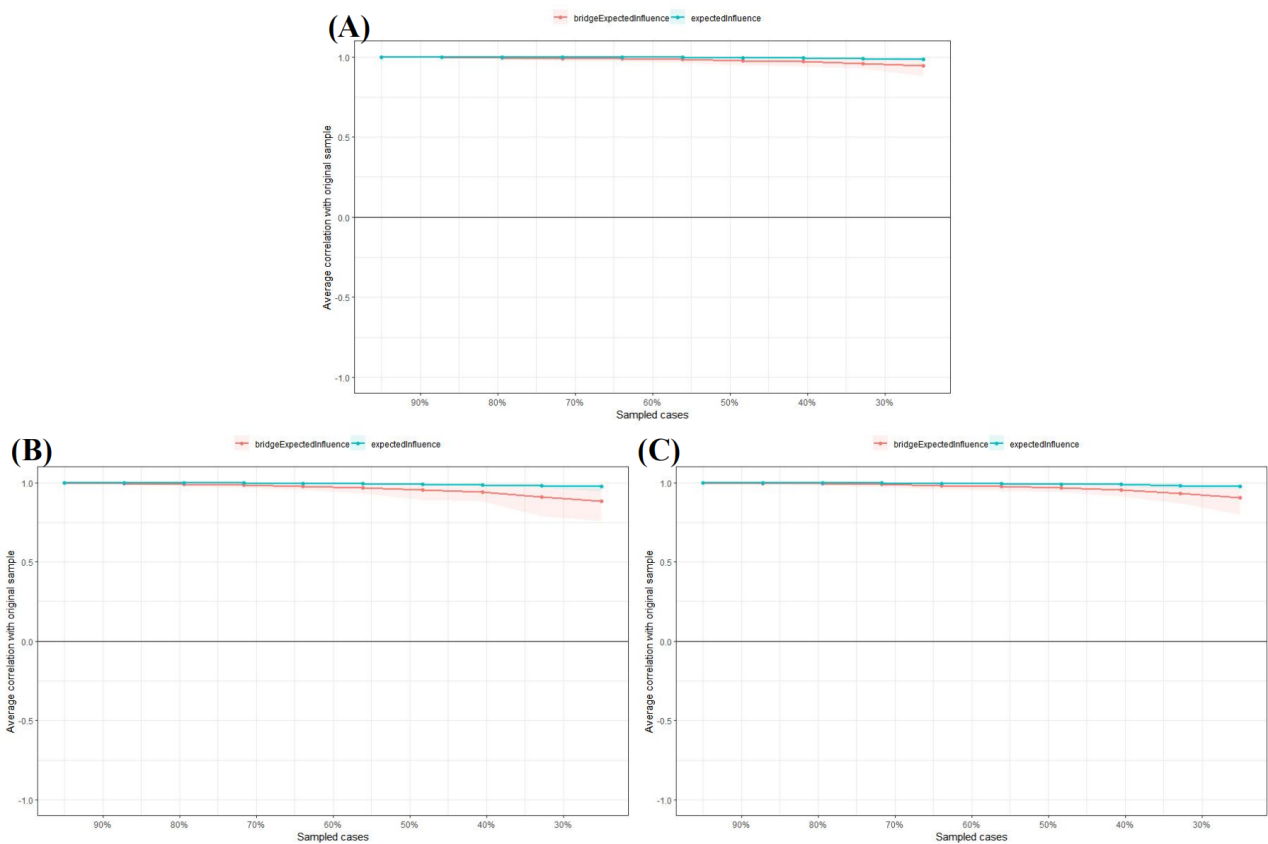


Figure S2. Stability estimations of expected influence and bridge expected influence using the case-drop bootstrapping method. (A) indicates all Chinese adolescents. (B) indicates male group. (C) indicate female group.

*Note:* The x-axis depicts the percentage of cases used at each step from the original sample. The y-axis shows the average of correlations between centrality indices in the original network and centrality indices in the re-estimated networks, after excluding increasing percentages of cases. The line represents the correlations of expected influences and bridge expected influences.


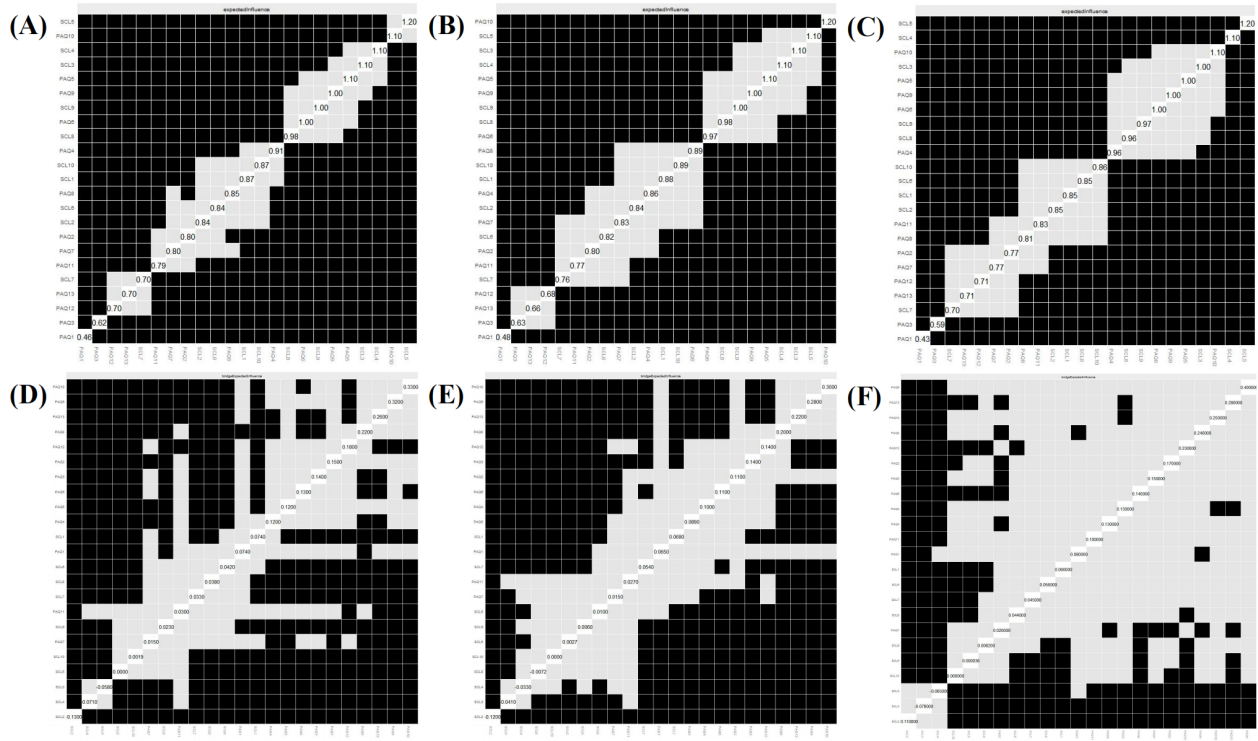


Figure S3. Nonparametric bootstrapped difference test for EI or BEI. (A) indicates all Chinese adolescents for EI. (B) indicates male group for EI. (C) indicate female group for EI. (D) indicates all Chinese adolescents for BEI. (E) indicates male group for BEI. (F) indicate female group for BEI.

*Note:* The black grid indicates a significant difference between the two corresponding edge weights, and the gray grid indicates no significant difference. The number in the diagonal boxes represent each of the node’s EI or BEI.


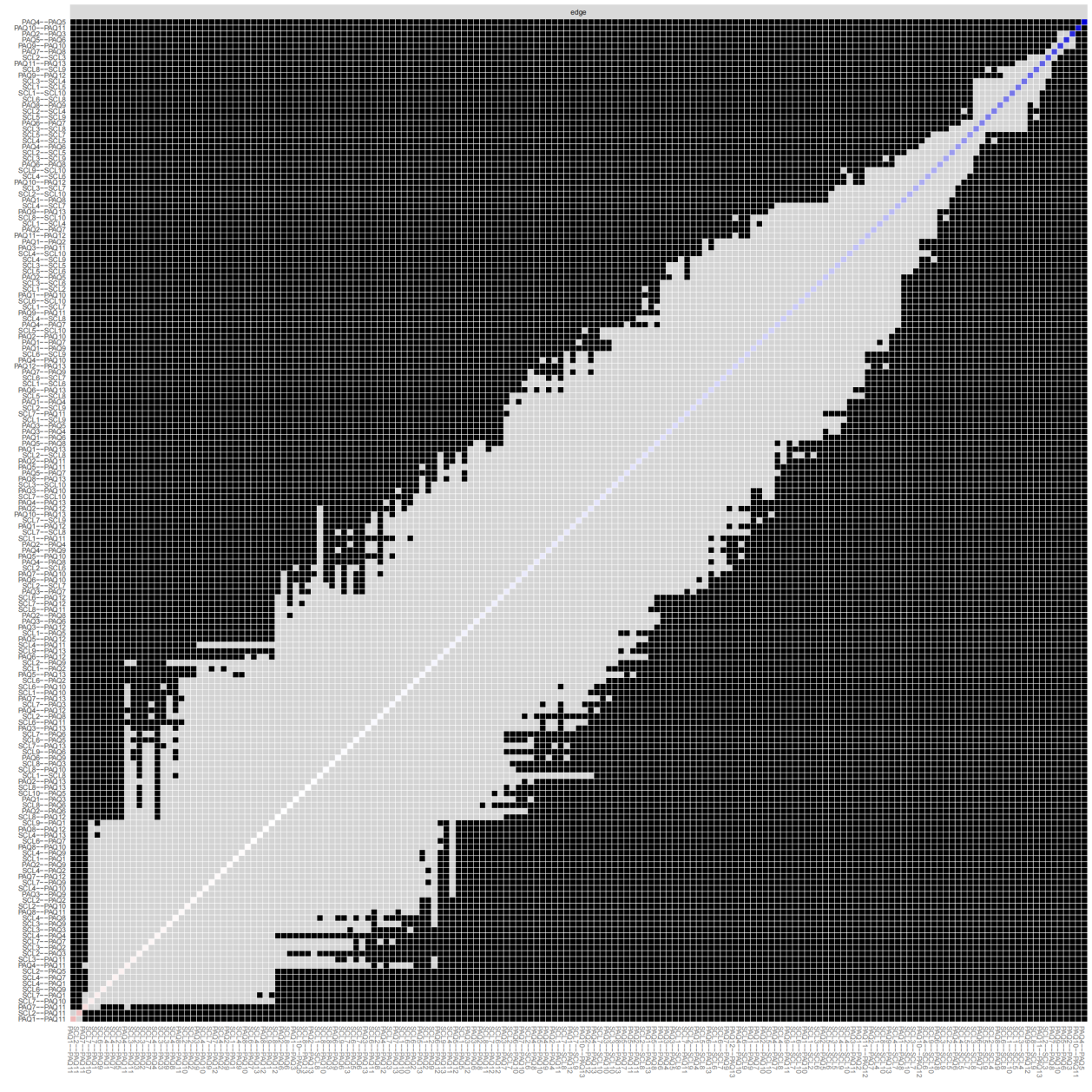


Figure S4. Nonparametric bootstrapped difference test for all edge weights in the Chinese adolescent network.

*Note:* Gray boxes indicate edge weights not significantly different from each other, while black boxes indicate significantly different edge weights. Blue and red boxes on the diagonals correspond to positively and negatively correlated edge weights, respectively.


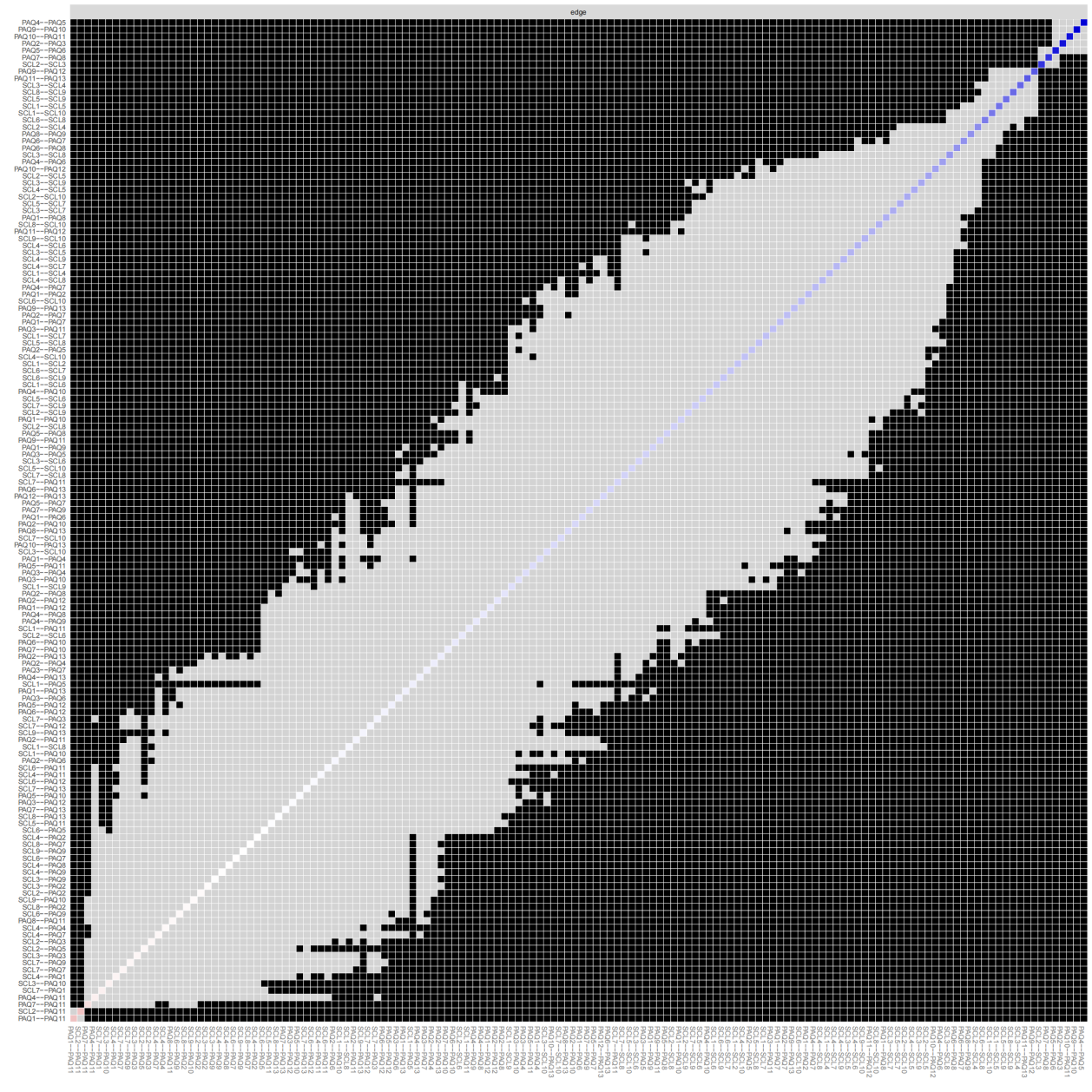


Figure S5. Nonparametric bootstrapped difference test for all edge weights in the male group’s network.

*Note:* Gray boxes indicate edge weights not significantly different from each other, while black boxes indicate significantly different edge weights. Blue and red boxes on the diagonals correspond to positively and negatively correlated edge weights, respectively.


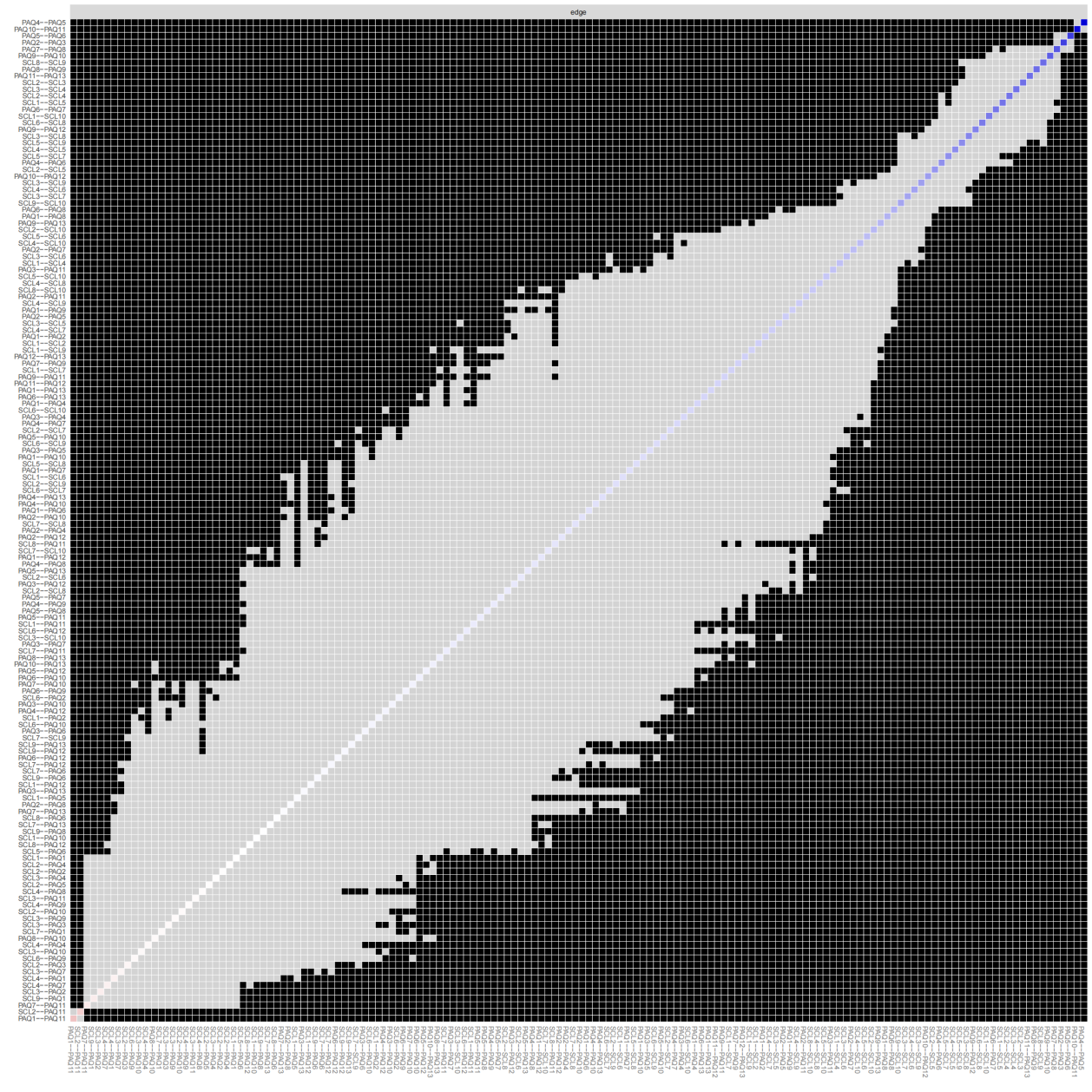


Figure S6. Nonparametric bootstrapped difference test for all edge weights in the female group’s network.

*Note:* Gray boxes indicate edge weights not significantly different from each other, while black boxes indicate significantly different edge weights. Blue and red boxes on the diagonals correspond to positively and negatively correlated edge weights, respectively.


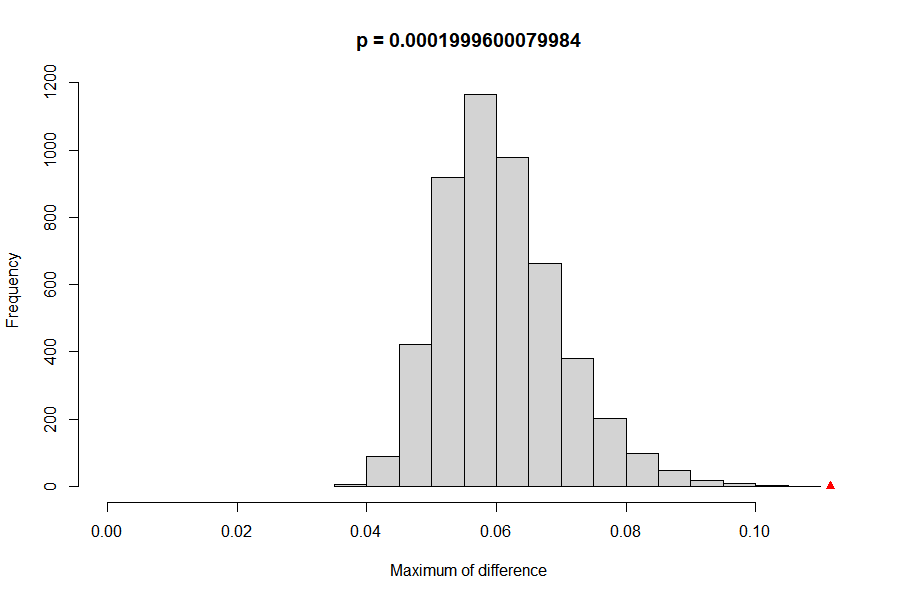


Figure S7. Bootstrapped value of the maximum difference in all of the edge weights by comparing the male network and the female network. (5000 permutations).

*Note:* The difference was significant (M=0.112, p=0.0002<0.001).


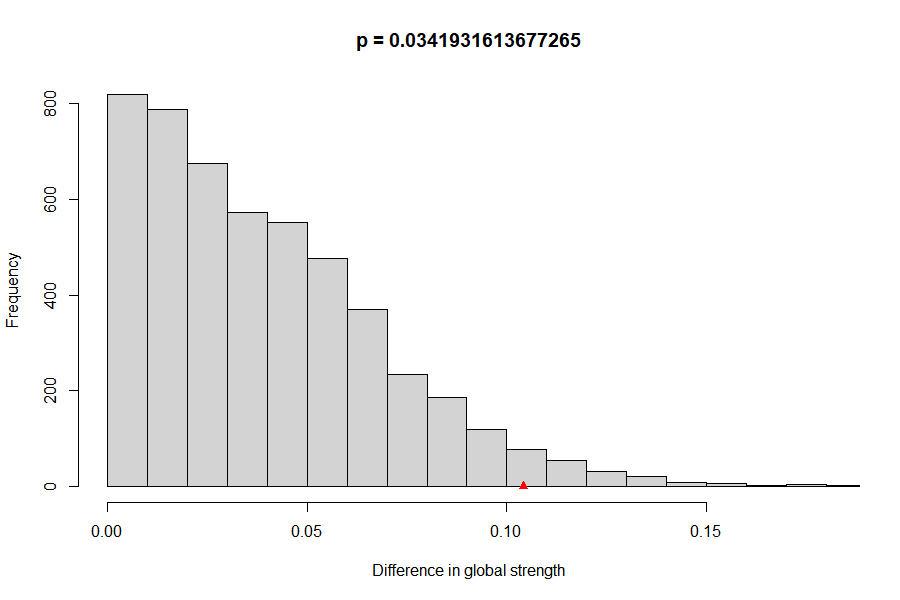


Figure S8. Bootstrapped value of the difference in network global strength by comparing the male network and the female network. (5000 permutations).

*Note:* The difference was significant (network strength among male participants: 10.154; among female participants: 10.050; p=0.036<0.05). Invariance in edge weights was tested using the permutation test, generating sets of p-values for each edge-edge comparison. Holm-Bonferroni corrected p-values were all >0.05, except psychosis and physical education (SCL9-PAQ1), psychosis and ball sports (SCL9-PAQ10), and fitness routine and ball sports (PAQ9-PAQ10).


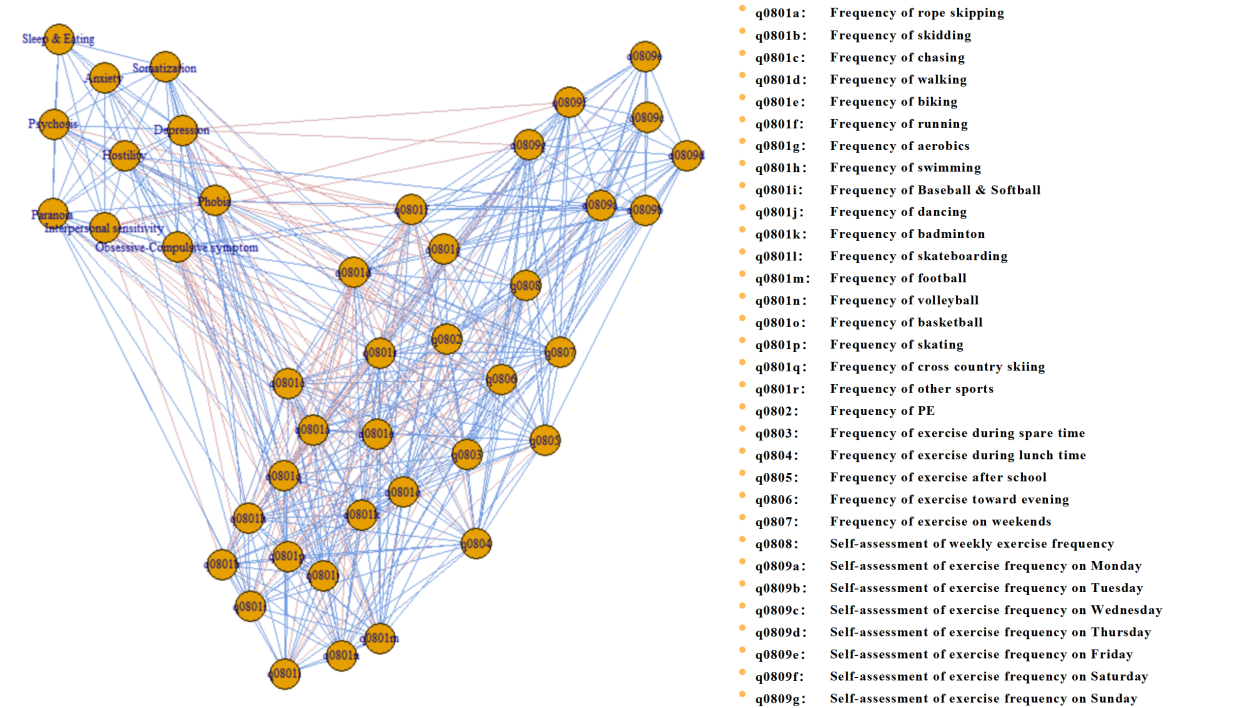


Figure S9. Network structures of all Chinese adolescents constructed by 10 SCL-90 nodes and 32 PAQ-A nodes.

*Note:* Blue edges represent positive associations, red edges indicate negative association. Larger widths of the edges indicating stronger correlations. The display mode of the picture is set as "Spring", divided into two societies, the upper left part represents the community of mental health symptoms, and the lower right part on behalf the physical exercise community. The specific meanings of the 32 PAQ-A nodes are listed on the right side of the figure.


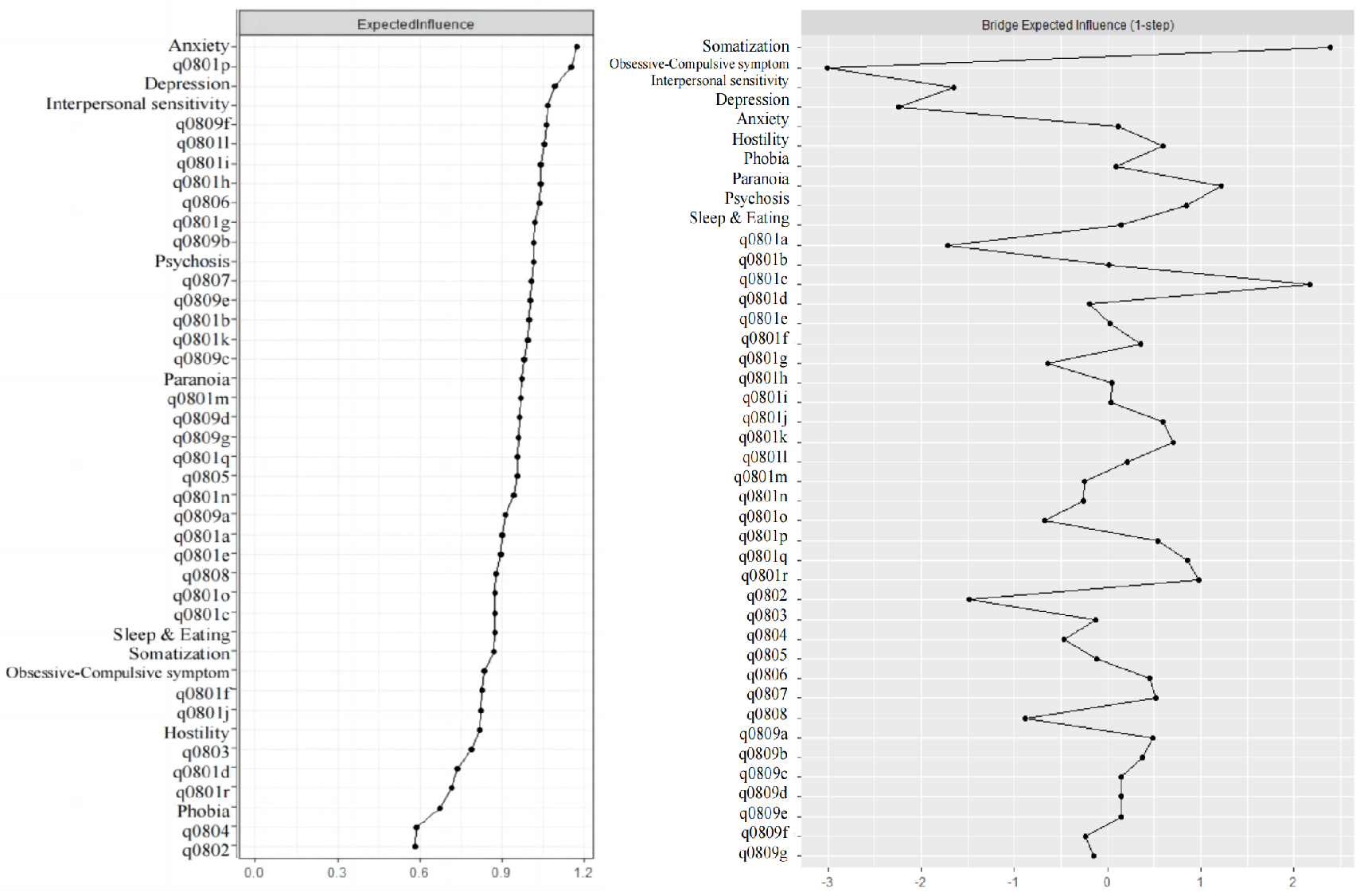


Figure S10. Centrality index based on the network constructed by 10 SCL-90 nodes and 32 PAQ-A nodes. (A) EI value of each node among all Chinese adolescents. (B) BEI value of each node among all Chinese adolescents.

*Note:* The chart on the left displays the EI scores, with anxiety having the highest score, followed by frequency of skating (q0801p), depression, interpersonal sensitivity, self-assessment of exercise frequency on Saturday (q0809f), frequency of skateboarding (q0801l), frequency of Baseball & Softball (q0801i), frequency of swimming (q0801h), and frequency of exercise after school (q0806). Therefore, these nine nodes are the core nodes for understanding the connection between mental health and physical exercise behavior in this network. The highest bridging nodes in the mental health community were obsessive-compulsive symptoms (negative) and somatization (positive). Frequency of rope skipping (q0801a) and physical education (q0802) were the highest negative bridging nodes in the physical exercise community. Furthermore, frequency of skidding (q0801b) and other sports (q0801r) were the most positively connected nodes in the physical exercise community. Without classifying 32 PAQ-A nodes, the results are similarly same as the network constructed by 10 SCL-90 nodes and 13 PAQ-A nodes.

Table S1 Values of edge weights between each of mental health symptoms and physical exercise characteristics among all Chinese adolescents.

|  | **SCL1** | **SCL2** | **SCL3** | **SCL4** | **SCL5** | **SCL6** | **SCL7** | **SCL8** | **SCL9** | **SCL10** | **PAQ1** | **PAQ2** | **PAQ3** | **PAQ4** | **PAQ5** | **PAQ6** | **PAQ7** | **PAQ8** | **PAQ9** | **PAQ10** | **PAQ11** | **PAQ12** | **PAQ13** |
| --- | --- | --- | --- | --- | --- | --- | --- | --- | --- | --- | --- | --- | --- | --- | --- | --- | --- | --- | --- | --- | --- | --- | --- |
| **SCL1** | 0.000 |  |  |  |  |  |  |  |  |  |  |  |  |  |  |  |  |  |  |  |  |  |  |
| **SCL2** | 0.081 | 0.000 |  |  |  |  |  |  |  |  |  |  |  |  |  |  |  |  |  |  |  |  |  |
| **SCL3** | 0.000 | 0.255 | 0.000 |  |  |  |  |  |  |  |  |  |  |  |  |  |  |  |  |  |  |  |  |
| **SCL4** | 0.101 | 0.194 | 0.213 | 0.000 |  |  |  |  |  |  |  |  |  |  |  |  |  |  |  |  |  |  |  |
| **SCL5** | 0.208 | 0.149 | 0.087 | 0.158 | 0.000 |  |  |  |  |  |  |  |  |  |  |  |  |  |  |  |  |  |  |
| **SCL6** | 0.064 | 0.033 | 0.083 | 0.125 | 0.086 | 0.000 |  |  |  |  |  |  |  |  |  |  |  |  |  |  |  |  |  |
| **SCL7** | 0.080 | 0.030 | 0.119 | 0.106 | 0.159 | 0.064 | 0.000 |  |  |  |  |  |  |  |  |  |  |  |  |  |  |  |  |
| **SCL8** | 0.004 | 0.048 | 0.181 | 0.079 | 0.063 | 0.199 | 0.035 | 0.000 |  |  |  |  |  |  |  |  |  |  |  |  |  |  |  |
| **SCL9** | 0.057 | 0.058 | 0.137 | 0.089 | 0.194 | 0.068 | 0.037 | 0.227 | 0.000 |  |  |  |  |  |  |  |  |  |  |  |  |  |  |
| **SCL10** | 0.203 | 0.118 | 0.041 | 0.091 | 0.072 | 0.081 | 0.040 | 0.102 | 0.125 | 0.000 |  |  |  |  |  |  |  |  |  |  |  |  |  |
| **PAQ1** | -0.006 | 0.000 | 0.000 | -0.022 | 0.000 | 0.000 | -0.027 | 0.000 | -0.001 | 0.000 | 0.000 |  |  |  |  |  |  |  |  |  |  |  |  |
| **PAQ2** | 0.014 | -0.009 | -0.015 | -0.007 | 0.000 | 0.013 | 0.000 | 0.000 | 0.000 | 0.000 | 0.092 | 0.000 |  |  |  |  |  |  |  |  |  |  |  |
| **PAQ3** | 0.000 | -0.017 | -0.013 | 0.000 | 0.000 | 0.000 | 0.010 | 0.005 | 0.000 | 0.000 | 0.002 | 0.318 | 0.000 |  |  |  |  |  |  |  |  |  |  |
| **PAQ4** | 0.000 | 0.000 | 0.000 | -0.013 | 0.000 | 0.000 | 0.000 | 0.000 | 0.000 | 0.000 | 0.058 | 0.034 | 0.056 | 0.000 |  |  |  |  |  |  |  |  |  |
| **PAQ5** | 0.021 | -0.020 | 0.000 | 0.000 | 0.000 | 0.007 | 0.000 | 0.000 | 0.000 | 0.002 | 0.000 | 0.084 | 0.057 | 0.379 | 0.000 |  |  |  |  |  |  |  |  |
| **PAQ6** | 0.000 | 0.000 | 0.000 | 0.000 | 0.000 | 0.000 | 0.008 | 0.001 | 0.006 | 0.000 | 0.053 | 0.001 | 0.023 | 0.153 | 0.314 | 0.000 |  |  |  |  |  |  |  |
| **PAQ7** | 0.000 | 0.000 | 0.000 | -0.021 | 0.000 | -0.003 | -0.014 | 0.000 | 0.000 | 0.000 | 0.070 | 0.101 | 0.030 | 0.077 | 0.044 | 0.187 | 0.000 |  |  |  |  |  |  |
| **PAQ8** | 0.000 | 0.009 | 0.000 | -0.011 | 0.000 | 0.000 | 0.000 | 0.000 | 0.000 | 0.000 | 0.115 | 0.025 | 0.000 | 0.034 | 0.050 | 0.136 | 0.273 | 0.000 |  |  |  |  |  |
| **PAQ9** | 0.000 | 0.016 | -0.011 | -0.006 | 0.000 | -0.023 | -0.007 | 0.000 | 0.000 | 0.000 | 0.070 | -0.006 | -0.008 | 0.034 | 0.000 | 0.006 | 0.066 | 0.195 | 0.000 |  |  |  |  |
| **PAQ10** | 0.011 | -0.010 | 0.000 | -0.008 | 0.000 | 0.013 | -0.027 | 0.004 | 0.000 | 0.000 | 0.081 | 0.071 | 0.040 | 0.067 | 0.034 | 0.031 | 0.033 | -0.005 | 0.292 | 0.000 |  |  |  |
| **PAQ11** | 0.034 | -0.095 | -0.019 | 0.019 | 0.000 | 0.009 | 0.058 | 0.025 | 0.000 | 0.000 | -0.107 | 0.046 | 0.092 | -0.019 | 0.045 | 0.000 | -0.049 | -0.010 | 0.079 | 0.350 | 0.000 |  |  |
| **PAQ12** | 0.000 | 0.000 | 0.000 | 0.000 | 0.000 | 0.026 | 0.026 | 0.000 | 0.000 | 0.000 | 0.036 | 0.037 | 0.021 | 0.009 | 0.020 | 0.018 | -0.007 | -0.003 | 0.225 | 0.123 | 0.098 | 0.000 |  |
| **PAQ13** | 0.000 | 0.000 | 0.000 | -0.003 | 0.000 | 0.000 | 0.006 | 0.002 | 0.018 | 0.000 | 0.049 | 0.003 | 0.009 | 0.040 | 0.014 | 0.063 | 0.011 | 0.044 | 0.103 | 0.037 | 0.235 | 0.066 | 0.000 |

Table S2 Values of edge weights between each of mental health symptoms and physical exercise characteristics among for male group.

|  | **SCL1** | **SCL2** | **SCL3** | **SCL4** | **SCL5** | **SCL6** | **SCL7** | **SCL8** | **SCL9** | **SCL10** | **PAQ1** | **PAQ2** | **PAQ3** | **PAQ4** | **PAQ5** | **PAQ6** | **PAQ7** | **PAQ8** | **PAQ9** | **PAQ10** | **PAQ11** | **PAQ12** | **PAQ13** |
| --- | --- | --- | --- | --- | --- | --- | --- | --- | --- | --- | --- | --- | --- | --- | --- | --- | --- | --- | --- | --- | --- | --- | --- |
| **SCL1** | 0.000 |  |  |  |  |  |  |  |  |  |  |  |  |  |  |  |  |  |  |  |  |  |  |
| **SCL2** | 0.086 | 0.000 |  |  |  |  |  |  |  |  |  |  |  |  |  |  |  |  |  |  |  |  |  |
| **SCL3** | 0.000 | 0.287 | 0.000 |  |  |  |  |  |  |  |  |  |  |  |  |  |  |  |  |  |  |  |  |
| **SCL4** | 0.103 | 0.163 | 0.216 | 0.000 |  |  |  |  |  |  |  |  |  |  |  |  |  |  |  |  |  |  |  |
| **SCL5** | 0.199 | 0.131 | 0.107 | 0.122 | 0.000 |  |  |  |  |  |  |  |  |  |  |  |  |  |  |  |  |  |  |
| **SCL6** | 0.080 | 0.033 | 0.064 | 0.108 | 0.075 | 0.000 |  |  |  |  |  |  |  |  |  |  |  |  |  |  |  |  |  |
| **SCL7** | 0.090 | 0.000 | 0.117 | 0.103 | 0.118 | 0.083 | 0.000 |  |  |  |  |  |  |  |  |  |  |  |  |  |  |  |  |
| **SCL8** | 0.014 | 0.068 | 0.148 | 0.100 | 0.089 | 0.186 | 0.063 | 0.000 |  |  |  |  |  |  |  |  |  |  |  |  |  |  |  |
| **SCL9** | 0.046 | 0.071 | 0.124 | 0.103 | 0.205 | 0.082 | 0.074 | 0.211 | 0.000 |  |  |  |  |  |  |  |  |  |  |  |  |  |  |
| **SCL10** | 0.192 | 0.121 | 0.053 | 0.088 | 0.064 | 0.095 | 0.054 | 0.112 | 0.108 | 0.000 |  |  |  |  |  |  |  |  |  |  |  |  |  |
| **PAQ1** | 0.000 | 0.000 | 0.000 | -0.016 | 0.000 | 0.000 | -0.022 | 0.000 | 0.000 | 0.000 | 0.000 |  |  |  |  |  |  |  |  |  |  |  |  |
| **PAQ2** | 0.000 | -0.007 | -0.005 | -0.001 | 0.000 | 0.000 | 0.000 | -0.009 | 0.000 | 0.000 | 0.098 | 0.000 |  |  |  |  |  |  |  |  |  |  |  |
| **PAQ3** | 0.000 | -0.013 | -0.013 | 0.000 | 0.000 | 0.000 | 0.017 | 0.000 | 0.000 | 0.000 | 0.000 | 0.334 | 0.000 |  |  |  |  |  |  |  |  |  |  |
| **PAQ4** | 0.000 | 0.000 | 0.000 | -0.010 | 0.000 | 0.000 | 0.000 | 0.000 | 0.000 | 0.000 | 0.053 | 0.025 | 0.049 | 0.000 |  |  |  |  |  |  |  |  |  |
| **PAQ5** | 0.022 | -0.013 | 0.000 | 0.000 | 0.000 | 0.001 | 0.000 | 0.000 | 0.000 | 0.000 | 0.000 | 0.088 | 0.065 | 0.360 | 0.000 |  |  |  |  |  |  |  |  |
| **PAQ6** | 0.000 | 0.000 | 0.000 | 0.000 | 0.000 | 0.000 | 0.000 | 0.000 | 0.000 | 0.000 | 0.057 | 0.012 | 0.021 | 0.135 | 0.326 | 0.000 |  |  |  |  |  |  |  |
| **PAQ7** | 0.000 | 0.000 | 0.000 | -0.011 | 0.000 | -0.002 | -0.015 | -0.002 | 0.000 | 0.000 | 0.093 | 0.094 | 0.024 | 0.099 | 0.058 | 0.156 | 0.000 |  |  |  |  |  |  |
| **PAQ8** | 0.000 | 0.000 | 0.000 | -0.003 | 0.000 | 0.000 | 0.000 | 0.000 | 0.000 | 0.000 | 0.115 | 0.038 | 0.000 | 0.033 | 0.068 | 0.152 | 0.289 | 0.000 |  |  |  |  |  |
| **PAQ9** | 0.000 | 0.000 | -0.003 | -0.003 | 0.000 | -0.010 | -0.013 | 0.000 | -0.002 | 0.000 | 0.065 | 0.000 | 0.000 | 0.033 | 0.000 | 0.000 | 0.057 | 0.157 | 0.000 |  |  |  |  |
| **PAQ10** | 0.013 | 0.000 | -0.020 | 0.000 | 0.000 | 0.000 | 0.000 | 0.000 | -0.007 | 0.000 | 0.069 | 0.056 | 0.047 | 0.079 | 0.008 | 0.032 | 0.027 | 0.000 | 0.360 | 0.000 |  |  |  |
| **PAQ11** | 0.033 | -0.087 | 0.000 | 0.010 | 0.003 | 0.012 | 0.063 | 0.000 | 0.000 | 0.000 | -0.087 | 0.015 | 0.091 | -0.022 | 0.051 | 0.000 | -0.045 | -0.010 | 0.066 | 0.336 | 0.000 |  |  |
| **PAQ12** | 0.000 | 0.000 | 0.000 | 0.000 | 0.000 | 0.009 | 0.016 | 0.000 | 0.000 | 0.000 | 0.034 | 0.037 | 0.006 | 0.000 | 0.021 | 0.018 | 0.000 | 0.000 | 0.235 | 0.133 | 0.111 | 0.000 |  |
| **PAQ13** | 0.000 | 0.000 | 0.000 | 0.000 | 0.000 | 0.000 | 0.009 | 0.003 | 0.015 | 0.000 | 0.022 | 0.027 | 0.000 | 0.023 | 0.000 | 0.062 | 0.005 | 0.055 | 0.094 | 0.054 | 0.234 | 0.060 | 0.000 |

Table S3 Values of edge weights between each of mental health symptoms and physical exercise characteristics among for female group.

|  | **SCL1** | **SCL2** | **SCL3** | **SCL4** | **SCL5** | **SCL6** | **SCL7** | **SCL8** | **SCL9** | **SCL10** | **PAQ1** | **PAQ2** | **PAQ3** | **PAQ4** | **PAQ5** | **PAQ6** | **PAQ7** | **PAQ8** | **PAQ9** | **PAQ10** | **PAQ11** | **PAQ12** | **PAQ13** |
| --- | --- | --- | --- | --- | --- | --- | --- | --- | --- | --- | --- | --- | --- | --- | --- | --- | --- | --- | --- | --- | --- | --- | --- |
| **SCL1** | 0.000 |  |  |  |  |  |  |  |  |  |  |  |  |  |  |  |  |  |  |  |  |  |  |
| **SCL2** | 0.075 | 0.000 |  |  |  |  |  |  |  |  |  |  |  |  |  |  |  |  |  |  |  |  |  |
| **SCL3** | 0.000 | 0.221 | 0.000 |  |  |  |  |  |  |  |  |  |  |  |  |  |  |  |  |  |  |  |  |
| **SCL4** | 0.096 | 0.215 | 0.216 | 0.000 |  |  |  |  |  |  |  |  |  |  |  |  |  |  |  |  |  |  |  |
| **SCL5** | 0.213 | 0.161 | 0.076 | 0.176 | 0.000 |  |  |  |  |  |  |  |  |  |  |  |  |  |  |  |  |  |  |
| **SCL6** | 0.050 | 0.035 | 0.100 | 0.141 | 0.103 | 0.000 |  |  |  |  |  |  |  |  |  |  |  |  |  |  |  |  |  |
| **SCL7** | 0.070 | 0.055 | 0.139 | 0.076 | 0.171 | 0.049 | 0.000 |  |  |  |  |  |  |  |  |  |  |  |  |  |  |  |  |
| **SCL8** | 0.000 | 0.033 | 0.187 | 0.086 | 0.052 | 0.205 | 0.045 | 0.000 |  |  |  |  |  |  |  |  |  |  |  |  |  |  |  |
| **SCL9** | 0.072 | 0.050 | 0.147 | 0.080 | 0.178 | 0.055 | 0.014 | 0.225 | 0.000 |  |  |  |  |  |  |  |  |  |  |  |  |  |  |
| **SCL10** | 0.206 | 0.110 | 0.031 | 0.103 | 0.086 | 0.061 | 0.039 | 0.084 | 0.137 | 0.000 |  |  |  |  |  |  |  |  |  |  |  |  |  |
| **PAQ1** | -0.002 | 0.000 | 0.000 | -0.021 | 0.000 | 0.000 | -0.009 | 0.000 | -0.029 | 0.000 | 0.000 |  |  |  |  |  |  |  |  |  |  |  |  |
| **PAQ2** | 0.018 | -0.003 | -0.026 | 0.000 | 0.000 | 0.019 | 0.000 | 0.000 | 0.000 | 0.000 | 0.075 | 0.000 |  |  |  |  |  |  |  |  |  |  |  |
| **PAQ3** | 0.000 | -0.014 | -0.009 | 0.000 | 0.000 | 0.000 | 0.000 | 0.000 | 0.000 | 0.000 | 0.000 | 0.293 | 0.000 |  |  |  |  |  |  |  |  |  |  |
| **PAQ4** | 0.000 | -0.003 | -0.004 | -0.010 | 0.000 | 0.000 | 0.000 | 0.000 | 0.000 | 0.000 | 0.065 | 0.042 | 0.061 | 0.000 |  |  |  |  |  |  |  |  |  |
| **PAQ5** | 0.008 | -0.005 | 0.000 | 0.000 | 0.000 | 0.000 | 0.000 | 0.000 | 0.000 | 0.000 | 0.000 | 0.078 | 0.053 | 0.391 | 0.000 |  |  |  |  |  |  |  |  |
| **PAQ6** | 0.000 | 0.000 | 0.000 | 0.000 | 0.000 | 0.000 | 0.011 | 0.004 | 0.010 | 0.000 | 0.047 | 0.000 | 0.017 | 0.171 | 0.296 | 0.000 |  |  |  |  |  |  |  |
| **PAQ7** | 0.000 | 0.000 | -0.016 | -0.022 | 0.000 | 0.000 | 0.000 | 0.000 | 0.000 | 0.000 | 0.050 | 0.101 | 0.030 | 0.060 | 0.032 | 0.210 | 0.000 |  |  |  |  |  |  |
| **PAQ8** | 0.000 | 0.000 | 0.000 | -0.006 | 0.000 | 0.000 | 0.000 | 0.000 | 0.002 | 0.000 | 0.118 | 0.007 | 0.000 | 0.038 | 0.032 | 0.119 | 0.256 | 0.000 |  |  |  |  |  |
| **PAQ9** | 0.000 | 0.000 | -0.008 | -0.007 | 0.000 | -0.011 | 0.000 | 0.000 | 0.000 | 0.000 | 0.078 | 0.000 | 0.000 | 0.032 | 0.000 | 0.019 | 0.070 | 0.225 | 0.000 |  |  |  |  |
| **PAQ10** | 0.001 | -0.007 | -0.010 | 0.000 | 0.000 | 0.017 | 0.000 | 0.000 | 0.000 | 0.000 | 0.052 | 0.046 | 0.018 | 0.047 | 0.055 | 0.023 | 0.019 | -0.009 | 0.248 | 0.000 |  |  |  |
| **PAQ11** | 0.031 | -0.074 | -0.006 | 0.000 | 0.000 | 0.000 | 0.029 | 0.039 | 0.000 | 0.000 | -0.093 | 0.081 | 0.094 | 0.000 | 0.031 | 0.000 | -0.032 | 0.000 | 0.067 | 0.376 | 0.000 |  |  |
| **PAQ12** | 0.010 | 0.000 | 0.000 | 0.000 | 0.000 | 0.031 | 0.012 | 0.001 | 0.012 | 0.000 | 0.038 | 0.040 | 0.035 | 0.018 | 0.023 | 0.012 | 0.000 | 0.000 | 0.192 | 0.150 | 0.066 | 0.000 |  |
| **PAQ13** | 0.000 | 0.000 | 0.000 | 0.000 | 0.000 | 0.000 | 0.003 | 0.000 | 0.013 | 0.000 | 0.066 | 0.000 | 0.010 | 0.049 | 0.037 | 0.065 | 0.007 | 0.028 | 0.112 | 0.025 | 0.222 | 0.072 | 0.000 |
